# MET inhibitors in advanced non-small-cell lung cancer: a meta-analysis and review

**DOI:** 10.18632/oncotarget.20824

**Published:** 2017-09-11

**Authors:** Jung Han Kim, Hyeong Su Kim, Bum Jun Kim

**Affiliations:** ^1^ Division of Hemato-Oncology, Department of Internal Medicine, Kangnam Sacred-Heart Hospital, Hallym University Medical Center, Hallym University College of Medicine, Seoul 07441, Republic of Korea; ^2^ Department of Internal Medicine, National Army Capital Hospital, The Armed Forces Medical Command, Sungnam 13574, Republic of Korea

**Keywords:** MET, MET inhibitor, non-small-cell lung cancer, meta-analysis

## Abstract

The alterations of *MET* have been detected in non-small-cell lung cancer (NSCLC). However, survival benefit of MET inhibitors remains controversial. We performed this meta-analysis to evaluate the survival benefit of MET inhibitors combined with an epidermal growth factor receptor tyrosine kinase inhibitor (EGFR-TKI) or standard chemotherapy in patients with advanced or metastatic NSCLC. A systematic computerized search of the electronic databases was carried out. From seven studies, 2,577 patients were included in the meta-analysis. Compared with patients in the placebo group, patients who received an additional MET inhibitor did not show significantly improved progression-free survival (hazard ration (HR) = 0.92 [95% confidence interval (CI): 0.79–1.08], *P* = 0.33) and overall survival (HR = 1.0 [95% CI: 0.90–1.11], *P* = 0.97). In the subgroup analysis, patients with MET-high NSCLC tended to show longer survival when treated with an additional MET inhibitor than those in the placebo group (HR = 0.76, [95% CI: 0.58–1.01], *P* = 0.06). In conclusion, this meta-analysis indicates that the addition of a MET inhibitor to an EGFR TKI or chemotherapy has no survival benefit over placebo in patients with advanced or metastatic NSCLC. Although patients with MET-high tumor tended to show better survival, further studies to explore more specific biomarkers are warranted to identify ideal candidates for MET inhibitors in NSCLC.

## INTRODUCTION

Lung cancer is the leading cause of cancer-related death worldwide [1, 2]. Most patients have advanced disease at diagnosis. For patients with advanced and metastatic non-small-cell lung cancer (NSCLC), systemic chemotherapy provides a modest but significant improvement in survival [3]. Treatment of NSCLC has progressed dramatically with the introduction of targeted agents over the last two decades. Recently immune checkpoint inhibitors emerged as a promising option in the fight against advanced NSCLC [4]. However, most tumors develop resistance to molecular targeted agents and their survival advantages are still disappointing. Therefore, there is a need to identify novel therapeutic targets promoting NSCLC pathogenesis and develop more efficacious targeted agents. MET/hepatocyte growth factor (HGF) pathway have recently emerged as a potential therapeutic target in various tumors including NSCLC [5, 6].

MET is the product of the proto-oncogene *MET* and the tyrosine kinase receptor for HGF [7]. The MET/HGF signaling pathway regulates multiple cellular functions, including differentiation, proliferation, and angiogenesis [5, 8]. Thus, dysregulation of the MET signaling pathway has been implicated in the pathogenesis of cancer, such as tumor cell proliferation and survival, invasion, and metastasis [9, 10]. In addition to gene amplification, mutations, or genetic polymorphisms, MET pathway can be activated protein overexpression by transcriptional up-regulation of *MET* or autocrine signaling of HGF [11, 12].

The enhanced expression of MET has been observed in a variety of malignancies [13–18]. Alterations in MET signaling have also been commonly observed in NSCLC [19]. High MET expression, HGF overexpression, or high *MET* gen copy numbers are associated with poor prognosis in patient with NSCLC [20–22]. The MET signaling pathway has cross-talks with the epidermal growth factor receptor (EGFR) network at both PI3K/Akt and MAPK nodes, suggesting mutual compensation [23]. *MET* activation has been proposed as a potential mode of resistance to EGFR tyrosine kinase inhibitors (EGFR-TKIs) in NSCLC [24–25]. Therefore, theoretically the combination of a MET inhibitor and an EGFR TKI may overcome this resistance [25–27]. Based on this scientific rationale, several MET inhibitors have been investigated in combination with EGFR TKIs or cytotoxic agents in NSCLC.

However, the survival benefits of MET inhibitors have not been consistent among studies in patients with NSCLC. Therefore, we performed this meta-analysis of randomized trials to evaluate the survival efficacy of MET inhibitors combined with an EGFR TKI or standard chemotherapy in patients with advanced or metastatic NSCLC.

## RESULTS

### Results of search

**Figure 1:**
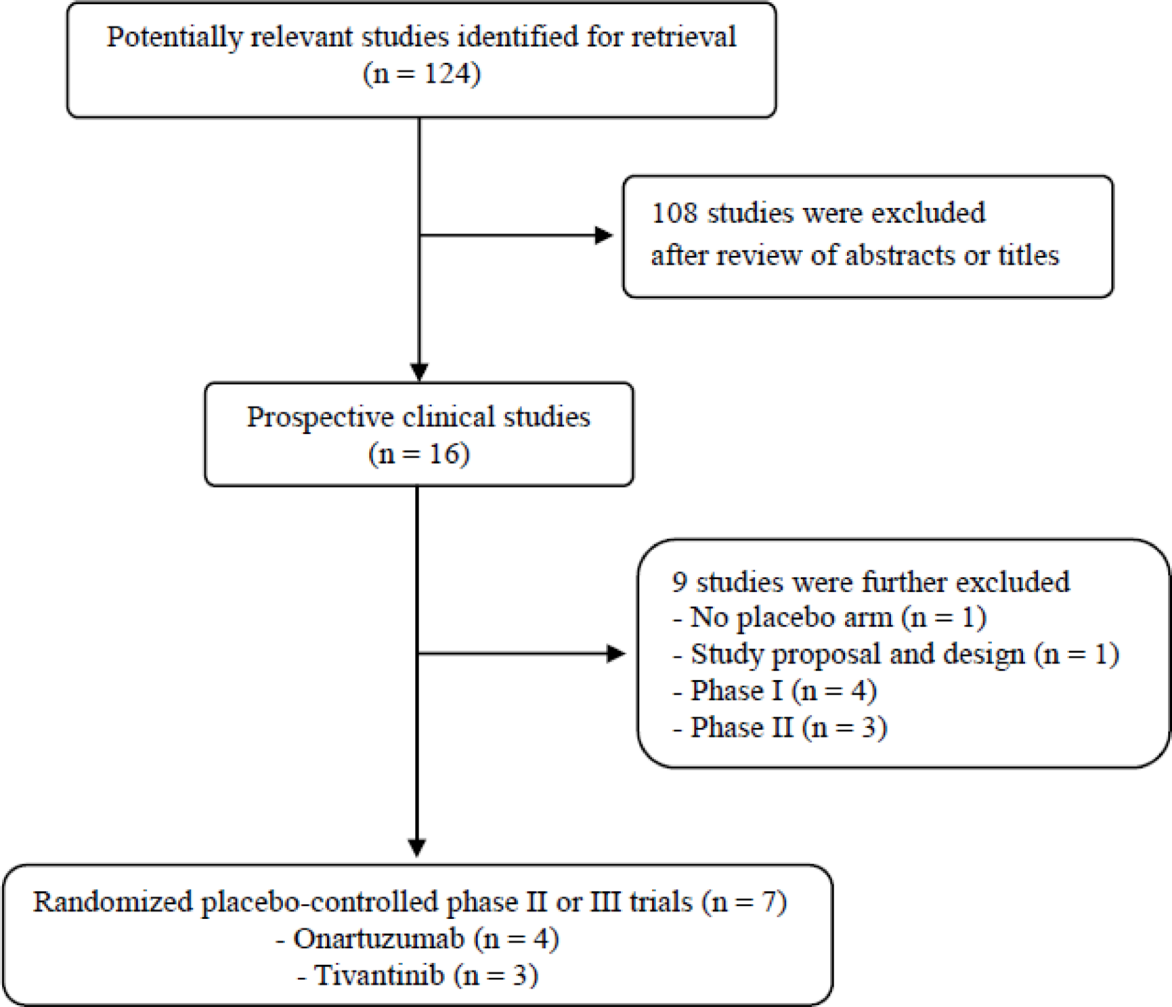
Flow diagram of search process

Figure 1 shows the flowchart of this meta-analysis. A total of 124 potentially relevant studies were initially found, but 108 of them were excluded after carefully screening the titles and abstracts. Of the remaining 16 potentially eligible studies, nine were further excluded by the inclusion criteria. Finally, seven studies were included in the meta-analysis [28–34].

### Characteristics of the included studies

Table 1 summarizes the main characteristics and clinical outcomes of the seven randomized phase II or III trials. Except for two [30, 31], five studies were conducted in patients with previously treated advanced or metastatic NSCLC [28, 29, 32–34]. Four trials used onartuzumab [28–31] and the remaining three used tivantinib [32–34]. There were no eligible randomized trials investigating the efficacy of other MET inhibitors including foretinib, cabozantinib, or amuvatinib. One study did not perform subgroup analysis according to the MET status [32]. The METLung study enrolled patients only with MET-high NSCLC [29] and the remaining five evaluated survival benefits in the subgroup with tumors showing high MET expression [28, 30–34]. All studies used immunohistochemistry (IHC) to determine MET status. One study had no criteria provided in the text [33], and five studies defined tumors with at least 50% of cancer cells stained positive with an intensity of 2+ or greater as high-MET or MET-positive [28–31, 34].

**Table 1:** Summary of the seven included studies

First author (yr) Study	Histology	Phase/ Setting	Arms (n)	Primary endpoint	No. of patients (high MET)	mPFS (mo) (high MET)	HR for PFS (95% CI)	mOS (mo) (high MET)	HR for OS (95% CI)
Spigel (2013) OAM4558g	NSCLC	II 2nd or 3rd	Erlotinib + Onartuzumab Erlotinib + Placebo	PFS	69 (31) 69 (35)	2.2 (2.9) 2.6 (1.5)	1.09 (0.73–1.62) *P* = 0.69 *0.53 (0.28–0.99) *P* = 0.04	8.9 (12.6) 7.4 (3.8)	0.80 (0.50–1.28) *P* = 0.34 *0.37 (0.19–0.72) *P* = 0.002
Spigel (2017) METLung	NSCLC	III 2nd or 3rd	Erlotinib + Onartuzumab Erlotinib + Placebo	OS	250 (250) 249 (249)	2.7 2.6	*0.99 (0.81–1.20) *P* = 0.92	6.8 9.1	*1.27 (0.98–1.65) *P* = 0.067
Hirsch (2016)	SQ	II 1st	Pac/Plat + Onartuzumab Pac/Plat + Placebo	PFS	55 (27) 54 (27)	4.9 (5.0) 4.9 (5.2)	0.95 (0.63–1.43) *P* = 0.80 *1.27 (0.69–2.32) *P* = 0.4405	9.1 (10.8) 8.5 (7.9)	0.90 (0.55–1.47) *P* = 0.68 *0.81 (0.40–1.64) *P* = 0.5485
Wakelee (2016)	Non-SQ	II 1st	Cohort 1 Bev/Pac/Plat + Onartuzumab Bev/Pac/Plat + Placebo Cohort 2 Pem/Plat + Onrtuzumab Pem/Plat + Placebo	PFS	69 (45) 70 (44) 59 (36) 61 (37)	5.0 (4.8) 6.8 (6.9) 4.9 (5.0) 5.1 (5.0)	1.25 (0.80–1.96) *P* = 0.333 *1.71 (0.97–3.02) 1.23 (0.81–1.86) *P* = 0.329 *1.25 (0.72–2.15)	NR (9.9) 16.5 (16.5) 8.5 (8.0) 13.7 (7.6)	1.34 (0.72–2.48) P = 0.352 *NA 1.15 (0.69–1.91) *P* = 0.591 *NA
Sequist (2011) ARQ 197–209	NSCLC	II 2nd or 3rd	Erlotinib + Tivantinib Erlotinib + Placebo	PFS	84 (NA) 83 (NA)	3.8 2.3	0.81 (0.57–1.16) *P* = 0.24 *NA	8.6 6.9	0.87 (0.59–1.27) *P* = 0.47 *NA
Yoshioka (2015) ATTENTION	Asian Non-SQ (*EGFR* WT)	III 2nd or 3rd	Erlotinib + Tivantinib Erlotinib + Placebo	OS	154 (77) 153 (83)	2.9 (NA) 2.0 (NA)	0.72 (0.54–0.95) *P* = 0.019 *NA	12.7 (NA) 11.1 (NA)	0.89 (0.67–1.19) *P* = 0.427 * 0.83 (0.56–1.24)
Scagliotti (2015) MARQUEE	Non-SQ	III 2nd or 3rd	Erlotinib + Tivantinib Erlotinib + Placebo	OS	526 (104) 522 (107)	3.6 (3.7) 1.9 (1.9)	0.74 (0.64–0.85) *P* < 0.001 *0.72 (0.52–0.99) *P* = 0.01	8.5 (9.2) 7.8 (5.9)	0.98 (0.84–1.14) *P* = 0.81 *0.70 (0.49–1.01) *P* = 0.03

NSCLC, non-small-cell lung cancer; SQ, squamous; Bev, bevacizumab; Pac, paclitaxel; Plat, platinum; Pem, pemetrexed; mPFS, median progression-free survival; mOS, median overall survival; HR, hazard ratio; CI, confidence interval; NR, not reached; NA, not available

* High MET: at least 50% of tumor cells stained positive with an intensity of 2+ or greater.

### Survival benefits of MET inhibitors in the intent-to-treat population

**Figure 2:**
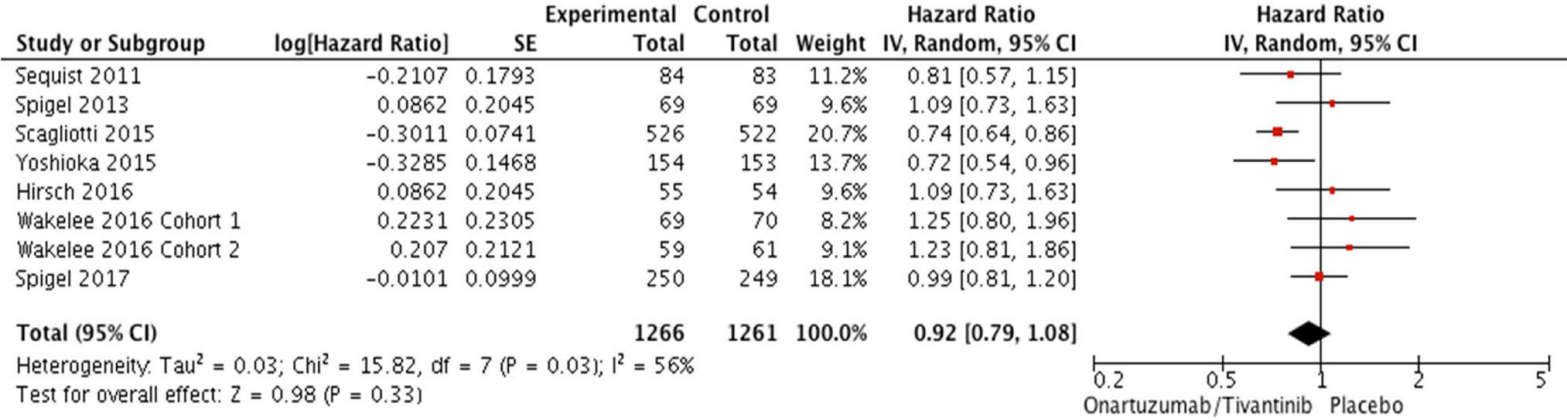
Forest plot of hazard ratios for progression-free survival

From the seven studies, a total of 2,577 patients were included in the meta-analysis of HRs for progression-free survival (PFS). Compared with the placebo, an additional MET inhibitor was not associated with significantly improved PFS (HR = 0.92 [95% confidence interval (CI): 0.79–1.08], *P* = 0.33) (Figure 2). There was a significant heterogeneity among studies (*X*^2^ = 15.82, *P* = 0.03, *I*^2^ = 56%) and the random-effects model was selected.

**Figure 3:**
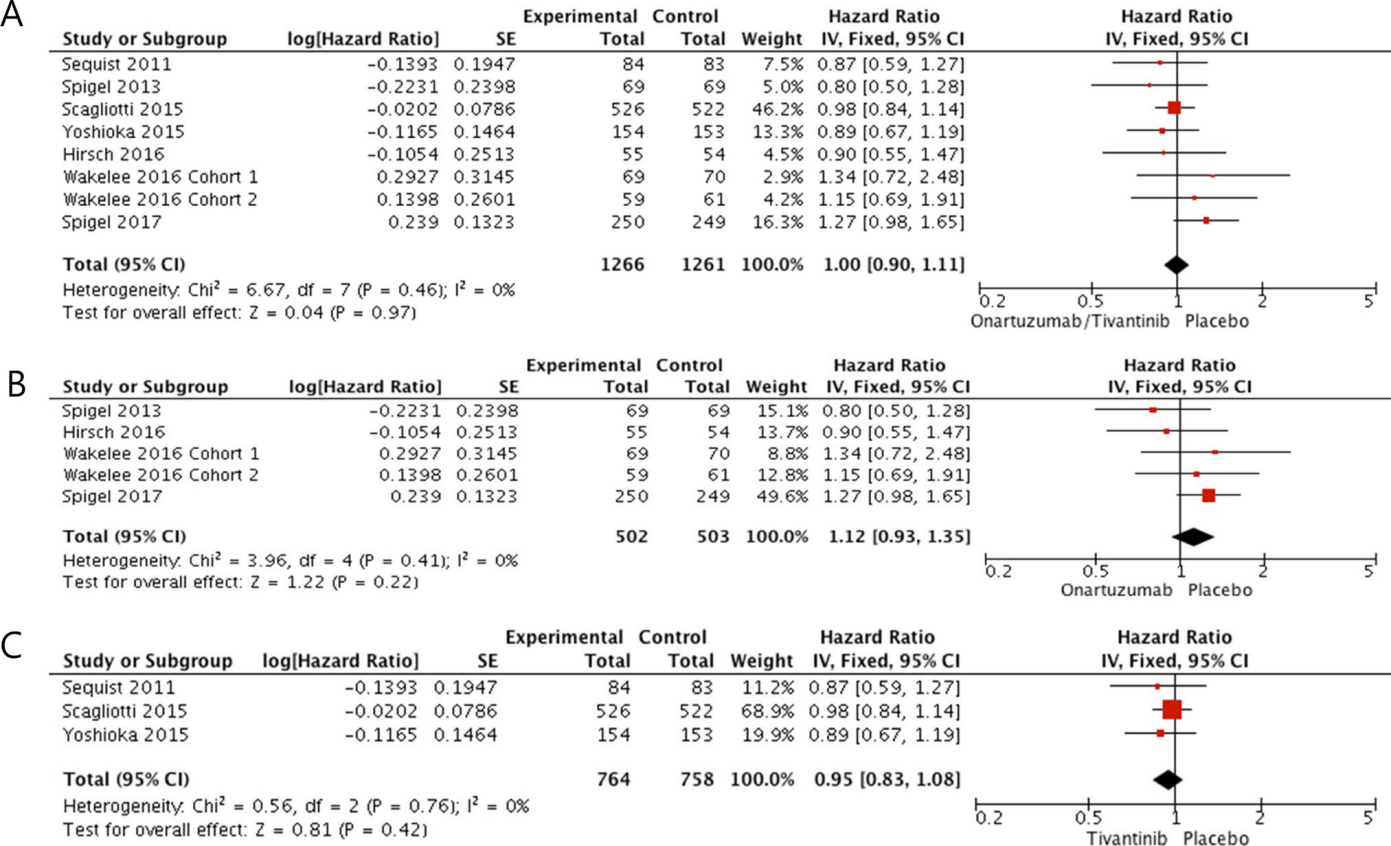
Forest plot of hazard ratios for overall survival **(A).** Subgroup analysis according to drug types: onartuzumab **(B)** and tivantinib **(C)**.

In terms of overall survival (OS), patients who received a MET inhibitor did not show survival benefit compared with those in the placebo group (HR = 1.0 [95% CI: 0.90–1.11], *P* = 0.97) (Figure 3A). There was no heterogeneity among studies (*X*^2^ = 6.67, *P* = 0.46, *I*^2^ = 0%) and the fixed-effects model was adopted. In the subgroup analysis according to drug types, neither onartuzumab (HR = 1.12, [95% CI: 0.93–1.35], *P* = 0.22) nor tivantinib (HR = 0.95, [95% CI: 0.83–1.08], *P* = 0.42) showed OS benefit when added to erlotinib or standard chemotherapy (Figure 3B and 3C).

### Survival benefits of MET inhibitors in the MET high subgroup

**Figure 4:**
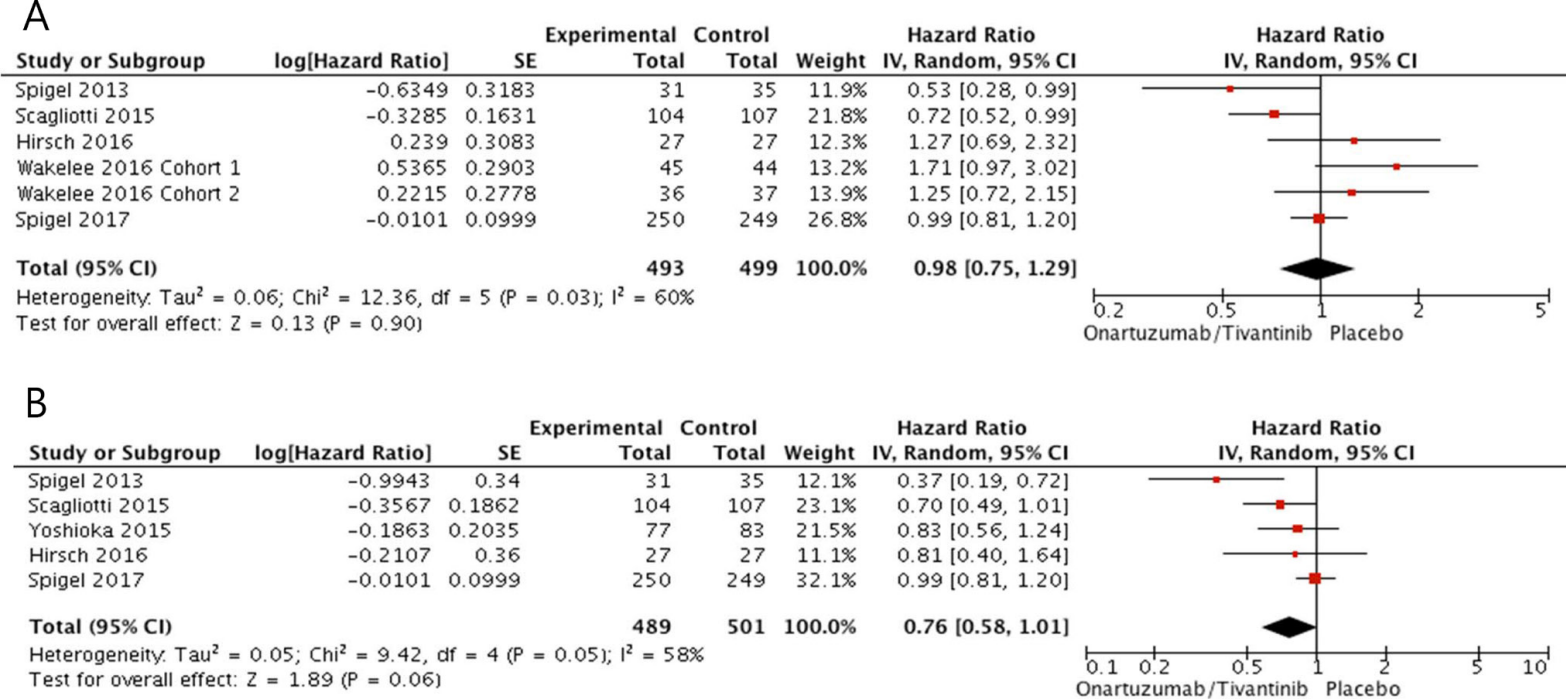
Forest plots of hazard ratios for progression-free survival (A) and overall survival (B) in patients with MET-high NSCLC

From five studies [28–31, 34], 992 patients with MET-high NSCLC were included in the meta-analysis of HRs for PFS. Patients treated with an additional MET inhibitor did not show better PFS compared with those in the placebo group (HR = 0.98 [95% CI: 0.75–1.29], *P* = 0.90) (Figure 4A). The random-effects model was applied because there was a significant heterogeneity across the studies (*X*^2^ = 12.36, *P* = 0.03, *I2* = 60%). In terms of OS, however, patients who received a MET inhibitor tended to show longer survival than those in the control group (HR = 0.76, [95% CI: 0.58–1.01], *P* = 0.06) (Figure 4B). The random-effects model was used because of a significant heterogeneity across the studies (*X*^2^ = 9.42, *P* = 0.05, *I*^2^ = 58%).

### Publication bias

**Figure 5:**
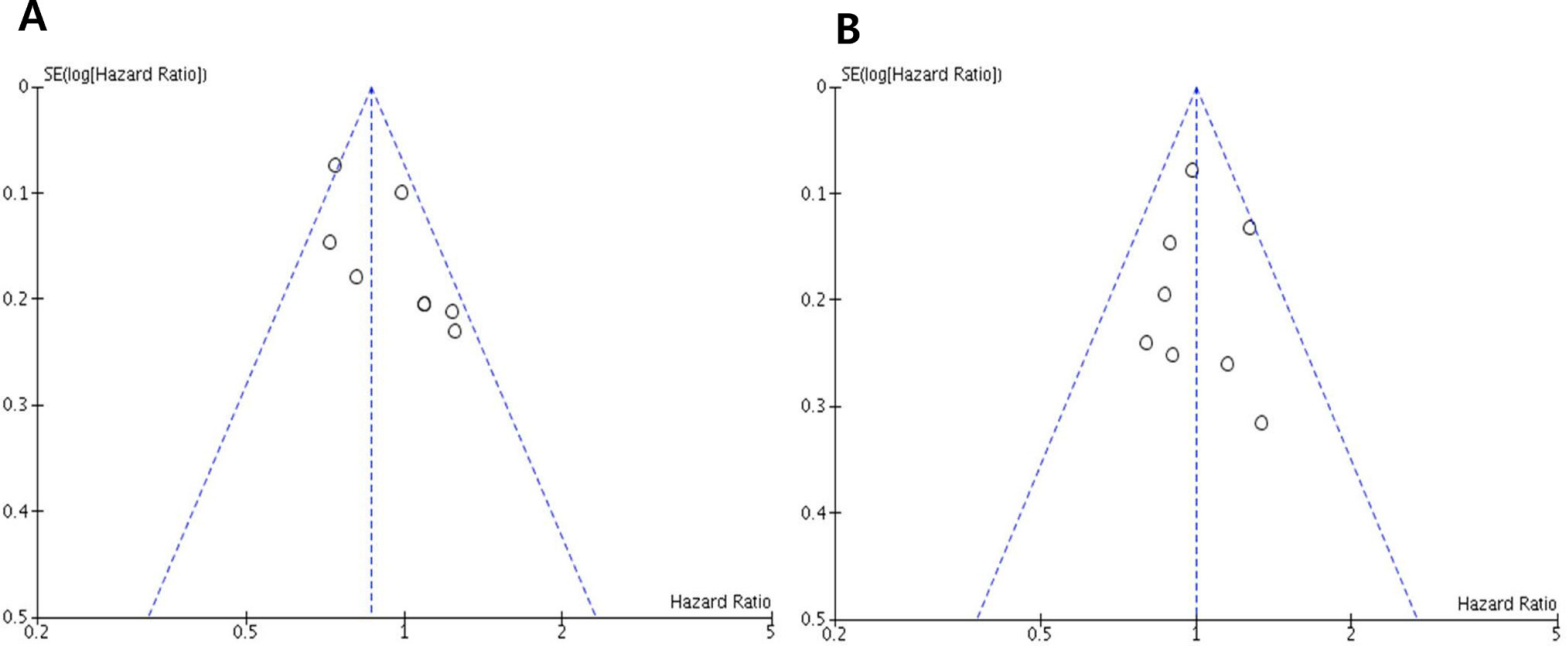
Funnel plots for publication bias regarding progression-free survival (A) and overall survival (B)

Visual inspection of the funnel plots for PFS and OS showed symmetry, indicating there were no substantial publication biases (Figure 5A and 5B).

## DISCUSSION

In this meta-analysis, we evaluated the survival efficacy of MET inhibitors (onartuzumab and tivantinib) in patients with advanced or metastatic NSCLC. The results indicate that the addition of a MET inhibitor to an EGFR TKI (erlotinib) or chemotherapy has no survival benefits over placebo in the intent-to-treat (ITT) population. To our knowledge, this is the first meta-analysis regarding the efficacy of MET inhibitors in patients with NSCLC.

There has been a strong rationale behind the trials of MET inhibitors in NSCLC. Increased MET signaling is linked to a worse prognosis for NSCLC [20–22]. A growing body of evidence has supported a link between the MET and EGFR signaling pathways [23]. Moreover, *MET* amplification has been associated with acquired resistance to EGFR-TKIs in *EGFR-*mutant NSCLC [24, 25]. Onartuzumab is a humanized monovalent monoclonal antibody that binds to the extracellular domain of MET protein to block HGF activation. In a randomized phase II trial (OAM4558g), combination of onartuzumab and erlotinib did not show any survival benefits in patients with previously treated advanced NSCLC [28]. However, onartuzumab plus erlotinib was associated with improved PFS (HR = 0.53, *P* = 0.04) and OS (HR = 0.37, *P* = 0.002) in patients with high level of MET expression, highlighting the importance of identifying biomarkers in drug development. Based on the results of this trial, the global phase III study (METLung) was conducted in patients with previously treated stage IIIB or IV MET-high (MET IHC 2+ or 3+) NSCLC [29]. Patients were randomized to receive a combination of either erlotinib plus onartuzumab or erlotinib plus placebo. The METLung study was stopped early at the time of the planned interim analysis with 499 patients enrolled due to the futility boundary crossed. The addition of onartuzumab to erlotinib did not prolong PFS (median 2.7 vs. 2.6 months, HR = 0.99, *P* = 0.92) and OS (median 9.1 vs. 8.5 months, HR = 1.27, *P* = 0.067). Subgroup analysis did not identify any prognostic factor to expect patients benefiting from addition onartuzumab to erlotinib. Based on promising early-phase results, two randomized phase II trials evaluated the efficacy of onartuzumab when added to first-line platinum-based chemotherapy for patients with squamous or non-squamous NSCLC [30, 31]. The results indicated that onartuzumab did not draw any clinical benefits from the addition to standard regimens, regardless of MET status.

Tivantinib is a selective, oral, non-ATP-competitive, small-molecule inhibitor of MET. A Western randomized phase II study (ARQ 197–209) investigated the clinical benefits of tivantinib combined with erlotinib in patients with previously treated NSCLC [32]. Although the study did not meet its primary endpoint (PFS, HR = 0.81, *P* = 0.24), the potential efficacy was demonstrated, especially among patients with *KRAS* mutations (HR for PFS = 0.18, *P* = 0.013). Then, two phase III trials (ATTENTION and MARQUEE) investigated the efficacy and safety of tivantinib combined with erlotinib in patients with previously treated advanced or metastatic non-squamous NSCLC [33, 34]. The Asian ATTENTION study was prematurely terminated due to the increased incidence of interstitial lung disease in the tivantinib group. Although the premature results showed that tivantinib plus erlotinib might improve PFS (HR = 0.72, *P* = 0.019), the study failed to improve OS (HR = 0.89, *P* = 0.427), regardless of MET status. The western MARQUEE study also failed to meet its primary endpoint of improved OS (HR = 0.98, *P* = 0.81), despite significant improvement in PFS (HR = 0.74, *P* < 0.001). Interestingly, however, the exploratory analysis indicated PFS (HR = 0.72, *P* = 0.01) and OS (HR = 0.70, *P* = 0.03) benefit with tivantinib in the subgroup of patients with MET-high status.

In the current meta-analysis of those randomized trials, the addition of a MET inhibitor to erlotinib or standard chemotherapy was not associated with significantly improved PFS (HR = 0.92, *P* = 0.33) and OS (HR = 1.0, *P* = 0.97) in the ITT population. Several plausible hypotheses may explain reasons for these negative results. First, although preclinical data have suggested the crosstalk between MET and EGFR [23], the role of *MET* amplification in the development of resistance to EGFR TKIs has been observed mostly in patients with *EGFR* mutation [24, 25, 35], not in patients with *EGFR* wild-type NSCLC. In our meta-analysis, patients with *EGFR* mutations in the experimental arm accounted for only about 10% of the patients tested. Thus, it is unlikely that this small proportion of patients would have driven OS benefit when treated with an additional MET inhibitor. Second, although activation of MET pathway can come from *MET* gen amplification or mutations [12], MET protein overexpression by transcriptional up-regulation of *MET* is the most frequent case in NSCLC. Therefore, there might be difference in the anti-tumor activity between MET monoclonal antibody and MET TKI. In the subgroup analysis, however, neither onartuzumab (HR = 1.12, *P* = 0.22) nor tivantinib (HR = 0.95, *P* = 0.42) showed OS benefit when added to erlotinib or standard chemotherapy. Third, MET protein overexpression might not be the best predictor for MET inhibitors in NSCLC. In the METLung study of NSCLC with high MET expression by IHC, the addition of onartuzumab to erlotinib failed to prolong OS over placebo (HR = 1.27, *P* = 0.067). In the subgroup analysis of our study, patients with MET-high NSCLC tended to show better OS (HR = 0.76, *P* = 0.06) when treated with an additional MET inhibitor. However, there is a need to identify more specific biomarkers for defining the subset of patients benefiting from MET inhibitors. MET overexpression is considered a late event consecutive to the transformed phenotype, deriving from transcriptional up-regulation of *MET* in absence of gene amplification or mutations or ligand-dependent autocrine or paracrine mechanism [11, 12, 36]. Therefore, targeting MET pathway in the tumors harboring MET overexpression as a late event would probably not draw a large survival benefit. The emerging data have suggested that splice-site mutations (*MET* exon 14) or high level of *MET* amplification lead to significant response to MET inhibitors [37–39]. Thus, *MET* mutation or *MET* true amplification may represent a better marker to select patients for MET inhibitors, especially MET TKIs.

Our study has several inherent limitations that need to be noted. First, the meta-analysis included a small number of studies. Second, the patients had different histological types (squamous or non-squamous) and received different combining agents (erlotinib or chemotherapeutic agents with/without bevacizumab) in various treatment settings (first-line or salvage setting). Third, one study had no IHC criteria for high MET expression. Finally, we did not include “crizotinib” which was initially characterized a TKI of MET in the search process. To our knowledge, however, there have been no randomized trials in patients with anaplastic lymphoma kinase (ALK)-negative NSCLC.

In conclusion, this meta-analysis indicates that the addition of a MET inhibitor to an EGFR TKI or standard chemotherapy has no survival benefits over placebo in patients with advanced or metastatic NSCLC. However, patients with MET-high tumor tended to show longer survival when treated with an additional MET inhibitor. Translational studies to explore more specific biomarkers are warranted to identify ideal candidates for MET inhibitors in NSCLC.

## MATERIALS AND METHODS

### Publication searching strategy

We performed this study according to the Preferred Reporting Items for Systematic Reviews and Meta-Analyses (PRISMA) guidelines [40]. A systematic computerized search of the electronic databases including PubMed, Embase, Google Scholar, and Cochrane Library (up to June 2017) was carried out. The search used the following keywords: “c-Met” or “MET,’ “onartuzumab,” “tivantinib,” “cabozantinib,” “foretinib,” “amuvatinib,” and “lung cancer.” We did not include “crizotinib” because it was approved only for the treatment of ALK-positive NSCLC. The related articles function in PubMed was used to identify all relevant articles.

### Inclusion criteria

Eligible studies should meet the following inclusion criteria: (i) studies were conducted in patients with NSCLC; (ii) randomized placebo-controlled trial; (iii) randomization of patients to systemic anti-cancer therapy (chemotherapy or targeted therapy) with or without a MET inhibitor. (iii) HRs and 95% CIs for PFS or OS were reported or could be estimated from the data provided; (iv) articles were written in English.

### Data extraction

Data extraction was carried out independently by two investigators (BJK and HSK). If these two authors did not agree, the principle investigator (JHK) was consulted to resolve the dispute.

The following data were extracted from the included studies: first author’s name, year of publication, number of patients, treatment arms, methodology to test MET status, the criteria used to dichotomize MET expression as ‘high (positive)’ or ‘low (negative)’, and HRs and their 95% CIs for PFS or OS.

### Statistical analysis

Statistical values were obtained directly from the original articles. When papers had no HRs and their 95% CIs, the Engauge Digitizer (version 9.1) was used to estimate the needed data from Kaplan-Meier curves. The effect size of PFS and OS was pooled through HR and its 95% CI. The heterogeneity across studies was estimated by using *Q* statistic and the *I*^2^ inconsistency test. The fixed-effects model (Mantel–Haenszel method) was selected for pooling homogeneous outcomes (*P* ≥ 0.1 and *I*^2^ *≤*  50%), and the random-effects model (DerSimonian–Laird method) was applied in the absence of significant heterogeneity (*P*   <  0.01 and *I*^2^ > 50%).

The RevMan version 5.2 was used to combine the data. The plots show a summary estimate of the results from all the studies combined. The size of the squares represents the estimate from each study, reflecting the statistical ‘weight’ of the study. Outcomes are provided as forest plots with diamonds representing the estimate of the pooled effect and the width of diamond representing its precision. The line of no effect is number one for binary outcomes, which depicts statistical significance if not crossed by the diamond [41]. All reported *P*-values were two-sided, with *P* < 0.05 defined as statistically significant. Publication bias was assessed graphically by the funnel plot method [42].
